# Revitalizing an important field in biophysics: The new frontiers of molecular crowding

**DOI:** 10.3389/fmolb.2023.1153996

**Published:** 2023-02-27

**Authors:** Marco Cammarata, Francesco Piazza, Germán Rivas, Giorgio Schirò, Piero Andrea Temussi, Annalisa Pastore

**Affiliations:** ^1^ Experiment Division, European Synchrotron Radiation Facility, 71 Ave des Martyrs, Grenoble, France; ^2^ Department of Physics & Astronomy, University of Florence and INFN Sezione di Firenze, Sesto Fiorentino, Italy; ^3^ Centro de Investigaciones Biológicas Margarita Salas, Consejo Superior de Investigaciones Científicas (CSIC), Madrid, Spain; ^4^ CNRS, CEA, IBS, University Grenoble Alpes, Grenoble, France; ^5^ Dipartimento di Scienze Chimiche, University “Federico II” of Naples, via Cynthia, Naples, Italy

**Keywords:** biophysics, biochemistry, phase separation, crowding, x-ray correlation spectroscopy, synchrotron radiation

## Abstract

Taking into account the presence of the crowded environment of a macromolecule has been an important goal of biology over the past 20 years. Molecular crowding affects the motions, stability and the kinetic behaviour of proteins. New powerful approaches have recently been developed to study molecular crowding, some of which make use of the synchrotron radiation light. The meeting “New Frontiers in Molecular Crowding” was organized in July 2022at the European Synchrotron Radiation facility of Grenoble to discuss the new frontiers of molecular crowding. The workshop brought together researchers from different disciplines to highlight the new developments of the field, including areas where new techniques allow the scientists to gain unprecedently expected information. A key conclusion of the meeting was the need to build an international and interdisciplinary research community through enhanced communication, resource-sharing, and educational initiatives that could let the molecular crowding field flourish further.

## Background of the crowding concept

**FIGURE 1:**
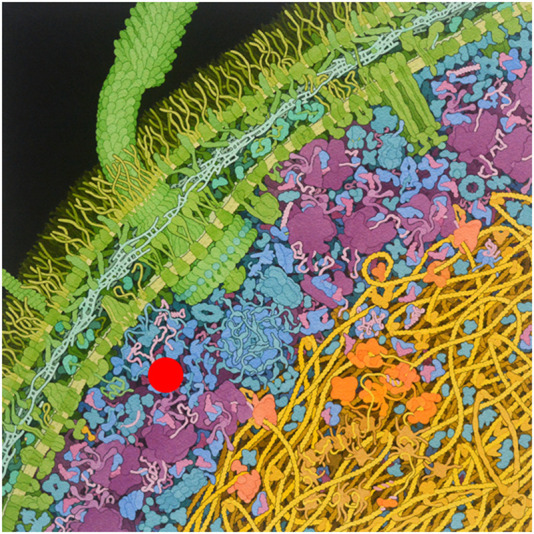
Cartoon of the crowded interior of an *Escherichia coli* cell. The test protein molecule (red) is in the cytoplasm exterior to the nucleoid, consisting of primarily soluble proteins, ribonucleic acids, and macromolecular assemblies such as ribosomes and proteasomes. Nonspecific interactions between the test protein and their immediate surroundings within a medium as heterogeneous and highly volume occupied as the bacterial cytoplasm can significantly influence the equilibria and rates of reactions (i.e., self- and hetero-associations) in which the test protein participates. Figure from Goodsell (http://mgl.scripps.edu/people/goodsell/illustration/public).

Classical *in vitro* biophysical studies have used dilute solutions to analyse properties of macromolecules from their stability to their diffusion constants. However, more realistic studies need to take into account the fact that the cell interior contains high amounts of macromolecules and, accordingly, it is usually described as “crowded” (Figure 1). The concept of crowding was originally introduced by Alexander George Ogston (Laurent and Ogston, 1963; Ogston, 1970) but it became popular only after Allen Minton published his first seminal articles on the subject (Minton, 1980; Minton, 1983; Minton, 2000).

Two different but not necessarily exclusive theories have been put forward to explain the effect of crowding: one based on a predominant entropic effect, the second based on enthalpy, as detailed in the following. To understand the role of crowding on protein behaviour, it was first assumed that prokaryotic cells could be considered as a “bag full of macromolecules”. According to this, the cytoplasm of *Escherichia coli* contains macromolecules in a range from 0.20 g/mL to 0.40 g/mL, that corresponds to up to 40% of the cellular volume (Zimmerman and Trach, 1991). As the result, the volume available to a small protein would be on average much lower than the total volume of the cell, if one assumes the principle of “excluded volume”, which means that two objects cannot occupy the same volume. A fundamental chemical consequence of volume exclusion is that it provides a generalized unspecific force to facilitate processes leading to a reduction in excluded volume, namely, macromolecular compaction and association (Minton, 2006). It has for instance been shown that crowding enhances reactions leading to the reduction of total excluded volume: crowding favors system states with smaller excluded volume, stabilizes compact macromolecular structures relative to expanded (unfolded) structures, enhances macromolecular associations in solution, enhances macromolecular affinity and decreases equilibrium solubility of macromolecules, thus enhancing the tendency to condense (Rivas and Minton, 2016). The expected magnitude of the effect is strongly dependent on the relative sizes and shapes of concentrated crowding species and on dilute macromolecular reactants and products (Alfano et al., 2017).

Although the view of cells as “bags full of proteins” is too simplistic, as questioned years ago by James Clegg (1984) and Srere, 2000, the concept of molecular crowding gained non-etheless widespread acceptance. The great majority of studies focused on the effect of crowding on protein stability, mainly because an early paper by Minton (2000) foresaw a large increase in melting temperatures, on the basis of an estimate of the volumes of folded and unfolded states of proteins. Conversely, most experimental studies showed only a modest increase in the temperature of unfolding (Theillet et al., 2014), suggesting that the influence of crowding on thermal stability is more complex than anticipated theoretically. In the attempt of explaining the discrepancy between the Minton’s model and the experimental results one possibility was to modify the premises, for instance, by abandoning the assumptions either of a large spherical shape of the unfolded state (Politou and Temussi, 2015), or of the exclusively entropic nature of crowding, i.e., the assumption that crowding effects are due only to hard-sphere interactions. The model of crowding introduced by Ogston (Laurent and Ogston, 1963; Ogston, 1970) and by Minton (Minton, 1980; Minton, 1983; Minton, 2000) is in fact essentially entropic, as clearly stated in the so-called depletion theory or Asakura-Oosawa model, put forward many years before the Minton model (Asakura and Oosawa, 1954; Asakura and Oosawa, 1958).

The concurrence or even the prevalence of enthalpic effects, other than volume exclusion, on the energetics and dynamics of macromolecular reactions in crowded environments have been raised since the first days of crowding studies (Miklos et al., 2010) and more recently has been investigated in more detail, both theoretically and experimentally (Speer et al., 2022). Several authors, mainly from the laboratory of Gary Pielak, noticed for instance that enthalpic contributions from some macromolecules could modulate protein stability, sometime leading to a decrease in stability (Miklos et al., 2010; Miklos et al., 2011; Wang et al., 2012; Sarkar et al., 2013; Senske et al., 2014; Cohen et al., 2015; Cohen and Pielak, 2016; Cohen and Pielak, 2017).

The work by these authors was essentially directed towards understanding the environment inside the cell, but it did not completely clarify the contribution of crowding on protein stability. It was already known that chemical interactions between proteins are of relevance, but it is difficult to study systems in which enthalpic effects are completely absent. What is possible is instead to minimize the enthalpic effects to evaluate the relative importance. This approach was attempted for instance by Alfano et al. (2017) who showed that the direct interactions of a few synthetic crowders with the Yfh1 protein, as observed from NMR spectra, are minimal. In these examples, the entropic effects in enhancing the protein stability could be separated from the enthalpic ones and clearly identified.

To discuss new approaches to study molecular crowding and put them into the context of the current field, we organized an international workshop on “New Frontiers in Molecular Crowding”. The workshop was strategically held at the European Synchrotron Radiation Facility (ESRF) of Grenoble (France) because this institution is currently the most advanced X-ray source that offers unique new possibilities to its users’ community. The aim of the workshop was to gather together experimentalists and computational researchers to discuss open questions in the biology and physics of molecular crowded systems. The program was centred on the role of the new possibilities offered by a variety of new advanced techniques, including those offered by synchrotron light sources, as well as other spectroscopic methods and theoretical simulations. In the last few years, the effect of crowded environments has in facts been widely studied using quite different techniques, ranging from fluorescence correlation spectroscopy to single particle tracking. Neutron and X-ray-based methods such as neutron spin echo (NSE) or X-ray Correlation Spectroscopy (XPCS) are particularly suited for in-cell studies as they are capable of characterizing diffusive processes over atomic distances. The promotion of these new possibilities is thus of primary importance. The integration of partly well established, partly emerging techniques in the study of crowded systems was discussed at the workshop with the aim of defining future applications.

The meeting brought together a group of ca. 50 theoreticians and experimentalists to exchange ideas on topics that could be grouped into three categories:

1) Theoretical predictions of the effects of excluded volume on the rates and/or equilibria of macromolecular reactions. As many aspects of the theory of crowding are already well developed, this opportunity for an interdisciplinary exchange came at a particularly appropriate time to involve a new community and favour cross-fertilization of the field.
2) The discussion on new experimental approaches that could be applied to the quantitative characterization of biochemical and biophysical processes in crowded media, both *in vitro* and *in vivo*.
3) Finally, a number of important examples and applications to specific biological phenomena in which crowding may play a major role. Special attention was paid into the study of phase separations and aggregation.

We will briefly review the major highlights of this important workshop as it may constitute a turning point in the crowding field of research.

## An overview of the Grenoble workshop

At the workshop, major theoretical contributions were given by a number of researchers who provided a vivid new perspective to the general concept of crowding. D. Harries of the Hebrew University in Jerusalem (Israel), for instance, discussed some of the experimental evidence to support the idea that crowding affects the equilibrium thermodynamics of protein stability and discussed the theories that have been put forward to explain these observations. He argued that most theories based on hard-core interactions predict crowding-induced entropic stabilization, whereas experimental evidence shows that crowding can also destabilize proteins (Sukenik et al., 2013; Sapir and Harries, 2015; Olgenblum et al., 2021; Speer et al., 2022). The speaker explained this discrepancy by the presence of chemical interactions between and among the protein, co-solutes, and water, i.e., enthalpic versus entropic effects.

A. Barducci from the INSERM-CNRS-UM in Montpellier (France) discussed the challenges associated with tackling problems of phase separations *via* computational methods, concluding that optimal combinations of multiscale coarse-grained (CG) models represent the most promising approaches to address this sort of questions in computer simulations. For example, he showed how 1-bead-per-domain CG models could be used to show that SIMs (SUMO Interacting Motifs) drive the assembly of a multifunctional scaffold protein that coordinates DNA. Along the same lines, Barducci introduced a 1-bead-per-protein coarse-grained approach to investigate how energy-consuming processes, such as phosphorylation, may play a crucial role in controlling the formation and stability of biomolecular condensates. In particular, the simulations revealed that phase separation raises concomitantly with the appearance of steady non-equilibrium mass currents between different phases, showing in a clear fashion that the energy-consuming reaction events happen more frequently at the interfaces between different phases. This is an important and general conclusion, which was also discussed by P. De Los Rios from EPFL, Lausanne (Switzerland), who provided an insightful discussion of the same problem, i.e., the role and nature of non-equilibrium steady fluxes in sustaining phase separation phenomena, based on an original analytical approach. Through reaction-diffusion calculations based on the Cahn-Hilliard formalism, De Los Rios showed that non-equilibrium reactions caused the presence of fluxes across the interfaces of condensates only in the presence of cyclic chemical reactions driven out of equilibrium.

J. Peters from the Institute Laue Langevin in Grenoble (France) presented the role of crowding under extreme conditions as low or high temperature, high hydrostatic pressure or salinity. This is an important topic for studying cells from extremophile bacteria that live under extreme conditions, both in their intact form and after cell lysis. She could distinguish the effect of crowding on the molecular dynamics of the proteome and of water molecules (Golub et al., 2018). She showed that crowding influences molecular dynamics and the environmental viscosity, having a protective effect against stress induced by extreme conditions.

F. Schreiber from University of Tübingen (Germany) discussed the implications of crowding for the phase behaviour focusing on the conditions of high concentration, with implications for aggregation phenomena, including clustering, crystallization, liquid-liquid phase transition, and various forms of arrested states. The speaker discussed the impact of temperature, salt and protein concentration, and other control parameters on the diffusion under equilibrium conditions, giving as examples aggregation phenomena, network formation upon denaturation, and phase separation.

A. Yethiraj of Memorial University of Newfoundland (Canada) examined a simple model system consisting of a probe polymer, polyethylene glycol, and a poly-sucrose crowder macromolecule (Ficoll-70), using NMR, rheology and small-angle neutron scattering. He found that, above a characteristic concentration, the polymers exhibit a universal exponential decrease in the translational diffusivity as a function of their concentration (Palit and Yethiraj, 2017). He then explored the nature of Ficoll and other well-known crowders, and showed that the excluded volume has been hugely underestimated. Ficoll for instance traps a quantity of bound water that is equal to or larger than its own weight. These contributions demonstrated that, despite the more than 20 years of research in crowding, there are still many aspects worth of being considered.

## Examples of molecular crowding in biological systems

A number of interesting biological systems were presented to provide direct examples of molecular crowding. Based on a combination of biophysical approaches, Chenal and coworkers from the Pasteur Institute in Paris (France) presented for instance how the structural flexibility of the adenylate cyclase toxin (CyaA) from *Bordetella pertussis* is essential for its secretion, folding, translocation across the plasma membrane, and intoxication of target cells. All these steps involve disorder-to-order structural transitions finely tuned by crowded environmental conditions that CyaA experiences during its life cycle. The researcher demonstrated the impact of crowding on some of these processes such as the calcium-induced folding upon toxin secretion.

M. Sagkiriadou from EPFL in Lausanne (Switzerland) presented a study of the dynamics and biophysical properties of the cytoplasm of the opportunistic pathogen *P. aeruginosa* under nitrogen starvation. He investigated the role of polyphosphate, a ubiquitous biological macromolecule that is important for bacterial stress response using fluorescence microscopy and particle tracking tools to probe the cellular interior. He found that polyphosphate plays an essential role in regulating the biophysical properties of the cytoplasm during starvation *via* various possible mechanisms.

J. Cecic Vidos and coworkers from the Centre de Biophysique Moléculaire in Orleans (France) examined the kinetics of enzymes located at the extracellular matrix, such as hyaluronidase, in the presence of crowders to mimic the natural environment in which these enzymes work. The speaker demonstrated how both size and concentration of crowders may influence enzymatic reactions.

A specifically important element of intracellular complexity is the presence of multiple microenvironments in which reactions may take place. These compartments add a different perspective to crowding, introducing the concept of confinement. While crowding and confinement are terms often used interchangeably, they have profound differences: crowding alludes to the effects imposed by the co-presence of many objects in a specific place and is a more dynamics term, whereas confinement, which refers to the limitations imposed by compartimentalization, is more static. Membrane-less organelles that cluster specific biomolecules away from the surrounding media represent one class of these microenvironments. They have been tentatively identified as immiscible liquid phases which arise through liquid-liquid phase separation (LLPS). The latter are also connected to the formation of biomolecular condensates, dynamic structures containing a wide range of protein and nucleic acids. Biomolecular condensation has emerged as a novel mechanism to organize intracellular biochemistry, with broad implications in physiology and pathology (Bai et al., 2022).

At the workshop, R. Winter from the Technical University of Dortmund (Germany) discussed the combined effects of temperature, pressure, osmolytes, macromolecular crowders and LLPS on the stability, conformational dynamics and reactivity of biomolecular systems. The speaker focused on protein stability, ligand binding, enzymatic activity and the conformational dynamics of non-canonical nucleic acids, such as DNA hairpins and G-quadruplexes.

G.G. Tartaglia from the Italian Institute of Technology of Genova (Italy) combined computational and experimental approaches to unravel LLPS association of non-coding RNAs (Cirillo et al., 2016) with proteins involved in transcriptional and translational regulation as well as neurodevelopmental (FXTAS) (Cid-Samper et al., 2018) and neurodegenerative diseases (Parkinson’s disease) (Marchese et al., 2016). By exploiting the finding that non-coding RNAs can act as scaffolds for protein-RNA interactions, he showed that specific properties of transcripts control the formation of large ribonucleic assemblies and their phase separation (Cerase et al., 2019).

S. Zorrilla and coworkers from CIB Margarita Salas CSIC, Madrid (Spain) analyzed two examples of reconstitution of bacterial cell division and their assembly in containers reproducing the crowded and phase-separated cell interior (Monterroso et al., 2021). In the first one, the partitioning of the protein FtsZ in an aqueous two-phase system mediated by non-associative LLPS revealed a differential partition pattern depending on the state of assembly of FtsZ linked to GTP binding and hydrolysis. In the second example, the FtsZ protein underwent associative LLPS when mixed with the DNA-binding nucleoid-associated SlmA protein (a negative modulator of FtsZ assembly at the division ring) under crowding conditions, producing condensates capable of reversible evolution to fibers in the presence of GTP.

Using in-cell folding experiments, S. Ebbinghaus from Technical University of Braunschweig (Germany) showed that the CAG RNA that is associated with Huntington disease, is largely destabilized in cells but remains folded in the cytoplasm, in the nucleolus and in condensates under physiological conditions. Upon temperature-induced unfolding, the RNA migrates into condensates, suggesting a preferential incorporation in its disordered state. The speaker also showed how ATP plays a crucial role to destabilize the CAG-hairpin in cells, by increasing the mobility of CAG RNA in condensates, and possibly preventing aberrant phase transitions that could be associated with nucleotide triplet-repeat expansion disease pathology.

E. Spruijt from Radboud University in Nijmegen (Netherlands) investigated the effects of macromolecular crowding on the formation and material properties of a model heterotypic biomolecular condensate, consisting of nucleophosmin (NPM1) and ribosomal RNA (rRNA). The speaker studied the effect of various macromolecular crowding agents, including PEG, dextran, Ficoll and cell lysates. He showed that PEG, usually assumed to be an inert crowding agent, can induce both homotypic and heterotypic phase separation of NPM1 and NPM1-rRNA.

G. De Luca from University of Palermo (Italy) presented a study of the aggregation process of human insulin under confined microscale aqueous compartments using steady state and time resolved linear and non-linear optical imaging. He showed that the compartment size affects the aggregation kinetics and the size of the resulting aggregates.

Y. Fichou of University of Bordeaux (France) combined biochemical assays and electron paramagnetic resonance spectroscopy (EPR) to study the properties of the tau protein under LLPS conditions to link LLPS with amyloid aggregation. He showed that tau LLPS relies on different types of hydrophobic and electrostatic interactions and that, depending on the forces, the protein properties in the dense phase are drastically different. Electrostatically-driven LLPS have little impact on tau dynamics and structure, and are independent of aggregation. Conversely, tau LLPS based on hydrophobic interactions triggers drastic modifications in the physics-chemical properties of the protein, resulting in the promotion of amyloid aggregation.

A. Morando from Fondazione Ri.MED in Palermo (Italy) presented data on the adhesion properties and coacervation of Pvfp-5β, one of the mussel foot proteins from the Asian green mussel *Perna viridis* thought to owe its properties thanks to post-translational modifications of the high number of Tyr in the sequence to 3, 4-dihydroxy-l-phenylalanine (DOPA) groups obtained by a post-translational modification of tyrosine. The speaker presented the structure and dynamics of Pvfp-5β in solution and demonstrated that the protein, previously considered intrinsically disordered, is instead stably folded, in agreement with the presence in the sequence of two EGF motifs (Santonocito et al., 2019; Morando et al., 2022). She then showed that the protein has adherent properties and coacervates also in the absence of DOPA modifications.

Taken together, all of these examples provided different perspectives into how crowding may affect aggregation and phase separation and underlined the importance of crowding in biologically relevant environments.

## New experimental and computational approaches for the study of molecular crowding

In view of the extreme complexity and heterogeneity of crowded environments, different experimental techniques have been proposed to characterize changes induced by molecular crowding ranging from anomalous diffusion (Höfling and Franosch, 2013) to stability (Stagg et al., 2007). Molecular diffusion has been studied with well-known techniques including single-molecular fluorescence and high-speed single particle imaging. While powerful, these techniques can only provide information on the hundreds of nanometre length scale at best, thus being unsuited for diffusion at the molecular length scale. Such limitation can be overcome using scattering techniques using sub-nm wavelengths.

**FIGURE 2:**
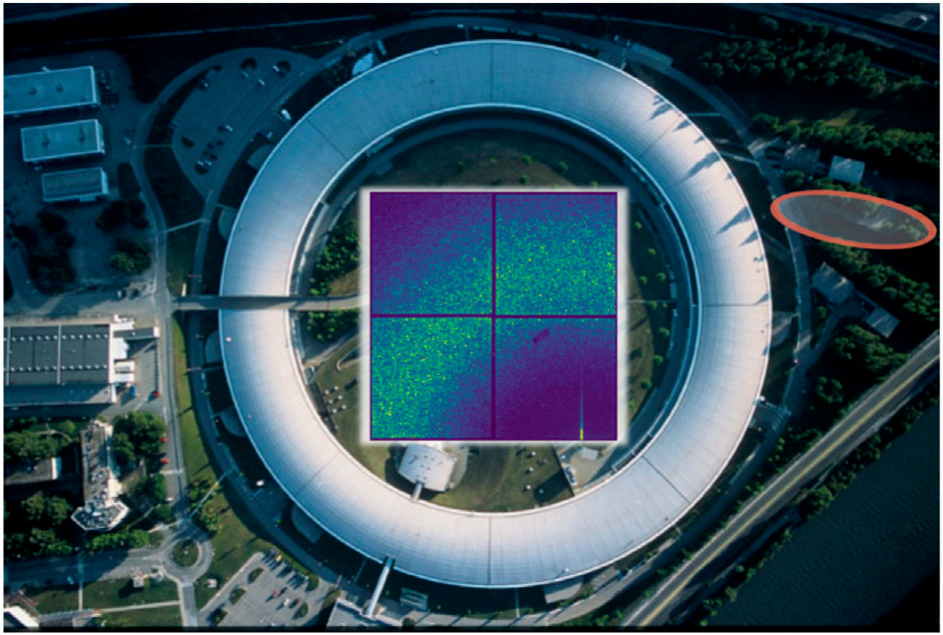
Aerial view of ESRF; the orange oval indicates the location of the new beamline devoted to the use of coherent x-rays currently under construction. Coherent x-rays have laser-like properties and produce speckle patterns when they illuminate disordered systems such as a protein solution. An example of speckle pattern is shown in the central part of the image. Since the speckle pattern encodes the position of each scatterer, they can be used to measure diffusion dynamics.

C. Gutt from the University of Siegen (Germany) discussed how the extension of the well-known light scattering in the X-ray regime can be used to probe dynamics down to the μs time scale but warned that care must be taken to avoid X-ray sample damage (Möller et al., 2019). Unsurprisingly, mitigation of these effects requires the use of a relatively large X-ray beam to decrease the X-ray dose. The large beam results in relatively small speckles (angular size of few μrad) that require large sample detector distance to be resolved with high contrast. Optimized optical components are also necessary to prepare the X-ray beam as discussed in the presentation of M. Cammarata’s from the ESRF in Grenoble (France). He presented the new X-ray beamline of ESRF devoted to XPCS and nanoscale imaging that is currently being built and that will become operational in 2025 (Figure 2). The new beamline will fully benefit from the recent ESRF storage ring upgrade that has boosted by ∼30 fold the coherence of the X-ray beam.

J. Möller from the European X-ray Free Electron Laser (Eu-XFEL) in Hamburg (Germany) showed how this facility provides unique opportunities for coherent scattering and imaging experiments by generating ultra-short, coherent hard X-ray pulses with megahertz repetition rate. The talk introduced the Materials Imaging and Dynamics (MID) instrument at Eu-XFEL (Madsen et al., 2021) and the different experimental possibilities that are offered for user experiments such as the MegaHertz X-ray Photon Correlation Spectroscopy (MHz-XPCS) that benefits from the highly coherent and pulsed X-ray radiation. The speaker showed recent experimental studies that demonstrate the possibility to study dynamics of biological samples at synchrotrons using ultra-low doses (Möller et al., 2019), reaching ns to sub-ps time resolution using split-and-delay optics at Eu-XFEL.

Using site-directed spin labeling coupled to electron paramagnetic resonance spectroscopy (SDSL-EPR), Mileo and co-workers from University of Marseille (France) investigated the structural dynamics of a cytosolic protein directly inside the cell combining nitroxide labels and EPR spectroscopy. They focused on the study of the structural organization of the NarJ protein both inside *E. coli* cells and in the presence of different crowders.

L. and A. Kedrov from the Heinrich Heine University in Duesseldorf (Germany) developed a membrane-adapted genetically encoded sensor protein that targets the membrane interface and is suitable for measuring interfacial crowding both *in vivo* and *in vitro* systems. The sensor consists of two fluorescent proteins forming a FRET pair, connected *via* a flexible linker and a hydrophobic transmembrane domain. Crowding induced by either proteins or polymers of varying sizes may be determined using the sensor, and the measurements may be carried out also in native cellular membranes, thus offering a robust approach for crowding analysis in complex environments.

G. Grassman from University of La Sapienza in Rome (Italy) proposed the combined use of extensive Molecular Dynamics (MD) simulations and a newly developed approach based on Zernike polynomials (Milanetti et al., 2020) to find complementary regions of interaction on the molecular surface and propose a set of possible association mechanisms using the RNA binding protein TDP-43 that is involved in amyotrophic lateral sclerosis.

## Future perspectives for the crowding community

The creation of a scientific community is a primary aspect of Science. Workshops and conferences are essential for the creation of such communities. The Grenoble workshop demonstrated the engagement of a large already existing but also in formation community who showed sincere enthusiasm for creating new collaborations and overtaking new important challenges. Taken together, the Grenoble workshop proved that many aspects both theoretical and experimental remain to be clarified and that, even more importantly, new tools may rapidly become a great asset to solve unresolved questions. In addition to crowding and partitioning of macromolecules between immiscible phases (LLPS and biomolecular condensation) other elements of the intracellular complexity need to be taken into account for a correct understanding of the energetics and dynamics of macromolecular reactions under crowding conditions such as those found inside living cells. Among them, it is worth mentioning the interfacial phenomena related to interactions between soluble macromolecules and surfaces.

Under this umbrella, the workshop was concluded by an inspirational short talk by G. Rivas from CIB Margarita Salas, CSIC, in Madrid (Spain), who reminded the participants of the importance to fill the gap between simplified models and cellular complexity (Rivas and Minton, 2018; Rivas and Minton, 2022). Interpreting the behaviour of proteins and macromolecular complexes inside the cell is very challenging due to the complexity and heterogeneity of the cellular system. Therefore, a possible strategy would be to design well-characterized reconstituted media incorporating one or more of the elements of the complexity of cells, whose composition can be controlled, to narrow the gap between the studies carried out *in vitro* and *in vivo*.

## Data Availability

The original contributions presented in the study are included in the article/supplementary material, further inquiries can be directed to the corresponding author.
